# Human Albumin Prevents 6-Hydroxydopamine-Induced Loss of Tyrosine Hydroxylase in *In Vitro* and *In Vivo*

**DOI:** 10.1371/journal.pone.0041226

**Published:** 2012-07-17

**Authors:** Li-Juan Zhang, Yue-Qiang Xue, Chun Yang, Wei-Hua Yang, Long Chen, Qian-Jin Zhang, Ting-Yu Qu, Shile Huang, Li-Ru Zhao, Xiao-Min Wang, Wei-Ming Duan

**Affiliations:** 1 Department of Anatomy, Capital Medical University, Beijing, China; 2 Department of Physiology, Capital Medical University, Beijing, China; 3 Department of Cellular Biology and Anatomy, Louisiana State University Health Sciences Center, Shreveport, Louisiana, United States of America; 4 Department of Biochemistry and Molecular Biology, Louisiana State University Health Sciences Center, Shreveport, Louisiana, United States of America; 5 Department of Biology, Xavier University of Louisiana, New Orleans, Louisiana, United States of America; 6 Department of Psychiatry, College of Medicine, University of Illinois at Chicago, Chicago, Illinois, United States of America; 7 Department of Neurology, Louisiana State University Health Sciences Center, Shreveport, Louisiana, United States of America; The Mental Health Research Institute of Victoria, The University of Melbourne, Australia

## Abstract

Human albumin has recently been demonstrated to protect brain neurons from injury in rat ischemic brain. However, there is no information available about whether human albumin can prevent loss of tyrosine hydroxylase (TH) expression of dopaminergic (DA) neurons induced by 6-hydroxydopamine (6-OHDA) toxicity that is most commonly used to create a rat model of Parkinson's disease (PD). In the present study, two microliters of 1.25% human albumin were stereotaxically injected into the right striatum of rats one day before or 7 days after the 6-OHDA lesion in the same side. *D*-Amphetamine-induced rotational asymmetry was measured 7 days, 3 and 10 weeks after 6-OHDA lesion. We observed that intrastriatal administration of human albumin significantly reduced the degree of rotational asymmetry. The number of TH-immunoreactive neurons present in the substantia nigra was greater in 6-OHDA lesioned rats following human albumin-treatment than non-human albumin treatment. TH-immunoreactivity in the 6-OHDA-lesioned striatum was also significantly increased in the human albumin-treated rats. To examine the mechanisms underlying the effects of human albumin, we challenged PC12 cells with 6-OHDA as an *in vitro* model of PD. Incubation with human albumin prevented 6-OHDA-induced reduction of cell viability in PC12 cell cultures, as measured by MTT assay. Furthermore, human albumin reduced 6-OHDA-induced formation of reactive oxygen species (ROS) and apoptosis in cultured PC12 cells, as assessed by flow cytometry. Western blot analysis showed that human albumin inhibited 6-OHDA-induced activation of JNK, c-Jun, ERK, and p38 mitogen-activated protein kinases (MAPK) signaling in PC12 cultures challenged with 6-OHDA. Human albumin may protect against 6-OHDA toxicity by influencing MAPK pathway followed by anti-ROS formation and anti-apoptosis.

## Introduction

Parkinson's disease (PD) is a debilitating neurodegenerative disorder characterized by the progressive loss of dopaminergic (DA) neurons in the substantia nigra pars compacta (SNc). Oral administration of levodopa remains the gold standard therapy for PD which is effective for symptomatic relief during early stage of PD. However, long-term administration of levodopa is always associated with side effects and less effectiveness. There is therefore a great need to develop a new therapy for PD. Although the mechanisms responsible for DA neuron death are not fully understood, accumulating evidence from both animal studies and human post-mortem studies suggests that oxidative stress plays the key role in initiating this cell death process [1]–[4]. The interventions into oxidative stress processes may potentially be developed into new therapeutic approaches for PD.

Neurotoxin induced-PD models are widely used to understand the mechanisms of neuronal degeneration in PD. 6-Hydroxydopamine (6-OHDA) is a selective catecholaminergic neurotoxin and is widely used both *in vivo* and *in vitro* studies to generate PD models. 6-OHDA is an analog of catecholamines that can be taken up by catecholaminergic terminals and is accumulated into cell bodies via the high-affinity retrograde transport system. After uptake, 6-OHDA is rapidly oxidized into the cytotoxic compounds 6-OHDA quinone and hydrogen peroxide and produces oxidative stress leading to cell death [5]. A growing number of reports suggest that auto-oxidation derived reactive oxygen species (ROS) [6], [7], NADPH oxidase-derived ROS [8], and early microglial activation [8], [9] play an essential role in 6-OHDA-induced cell death. However, the intrinsic molecular mechanisms of 6-OHDA-induced cytotoxicity are not fully elucidated.

It has been demonstrated that neuronal damage resulting from oxidative stress is related to the activation of stress-activated protein kinases in response to various stimuli [10]. A number of *in vivo* and *in vitro* studies have suggested that 6-OHDA-induced neuronal damage is associated with the activation of c-Jun N-terminal protein kinase (JNK) and p38 signaling pathway [11], [12]. Another member of mitogen-activated protein kinase (MAPKs), extracellular signal-regulated kinase (ERK), is also important for neuron survival [13].

Human albumin is produced by the liver. Human albumin can perform multiple biological functions including maintaining colloidal osmotic pressure, binding and transporting small molecules in the blood. Human albumin has been clinically used in serious and often life-threatening conditions, such as shock and blood loss due to trauma, burns, and surgery. Human albumin is also a potent antioxidant, acting as both a free radical scavenger and a chelator of transition metals and heme [14]. A recent series of studies has explored the efficacy of human albumin as a therapeutic agent in experimental models of stroke [15]–[18]. Although a number of possible mechanisms have been examined, including the effect of human albumin on local cerebral perfusion, blood-brain barrier disruption, systemic fatty acid responses, and microvascular patency [19], the mechanism underlying the neuroprotection produced by human albumin remains undetermined.

The present study was designed to examine the effects of human albumin on the TH expression of DA neurons after 6-OHDA toxin insult. In the first part of the study, human albumin was intrastriatally administered pre- or post-6-OHDA lesions to address whether human albumin could influence TH expression of DA neurons behaviorally and morphologically in a rat model of PD. In the second part of the study, exposure of rat pheochromocytoma (PC12) cells with 6-OHDA was used as a model system to examine the mechanisms underlying cytoprotection produced by human albumin. The cell viability, ROS formation, apoptosis and MAPK signaling were examined in cultured PC12 cells treated with and without human albumin.

## Results

### Intrastriatal injections of human albumin attenuate rotational asymmetry in a rat model of PD

**Figure 1:**
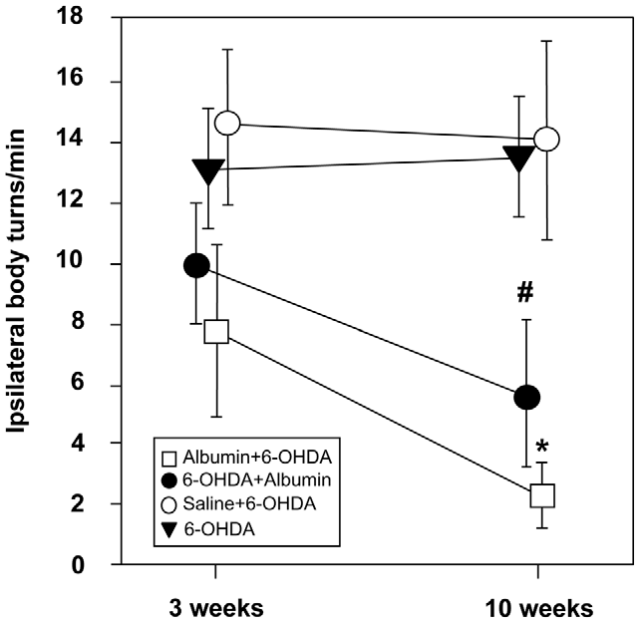
Intrastriatal administration of human albumin reduces d-amphetamine-induced rotational asymmetry in a rat model of PD. The diagram summarizes the scores of net rotational asymmetry in the Albumin +6-OHDA (n = 8), 6-OHDA +Albumin (n = 8), Saline +6-OHDA (n = 7) and 6-OHDA (n = 7) groups. Rats were unilaterally injected with 1.25% human albumin or saline 1 day before or 7 days after 6-OHDA lesion. Rotational behavior was assessed 3 weeks and 10 weeks after 6-OHDA lesion, respectively. The number of full 360° body turns to the ipsilateral direction was recorded for 90 min using an automated rotometer after rats were injected i.p. with 5 mg/kg *d*-amphetamine. The data are presented as mean values ± SEM. Two-factor ANOVA and one-factor ANOVA with post hoc comparison were applied. * *P*<0.01, # *P<*0.05 versus the Saline +6-OHDA and the 6-OHDA groups.

The *d*-amphetamine-induced rotation asymmetry scores were summarized in Fig. 1. A two-factor ANOVA did not reveal a significant group × time interaction of the net rotational scores over the testing period (*P*>0.05). There was no significant difference between any groups 3 weeks after 6-OHDA lesion (Albumin +6-OHDA group: 8±2.7, 6-OHDA + Albumin group: 10±2.0, Saline +6-OHDA group: 14±2.3, and 6-OHDA group: 13±1.6) (*P*>0.05). However, both pre- and post-treatment with human albumin resulted in a significant reduction of the number of *d*-amphetamine-induced rotations for hemiparkinsonian rats in the Albumin +6-OHDA group (2±1.0) and the 6-OHDA + Albumin group (6±2.5) 10 weeks after 6-OHDA lesion when compared with the Saline +6-OHDA group (14±2.8) and the 6-OHDA group (13±2.3) (*P<0.01, # *P*<0.05). The mean values of *d*-amphetamine-induced rotations appeared to be lower in the Albumin +6-OHDA group than that in the 6-OHDA + Albumin group at both 3 weeks and 10 weeks time-points. However, no significant difference was observed between the two groups at both time-points (*P*>0.05). Furthermore, no significant difference in rotational asymmetry was observed in the Saline +6-OHDA group and the 6-OHDA group 3 weeks and 10 weeks after 6-OHDA lesion (*P*>0.05).

### Intrastriatal injections of human albumin affect TH expression in DA neurons in the SNc following 6-OHDA-intoxication

#### TH-immunoreactivity in the striatum

**Figure 2:**
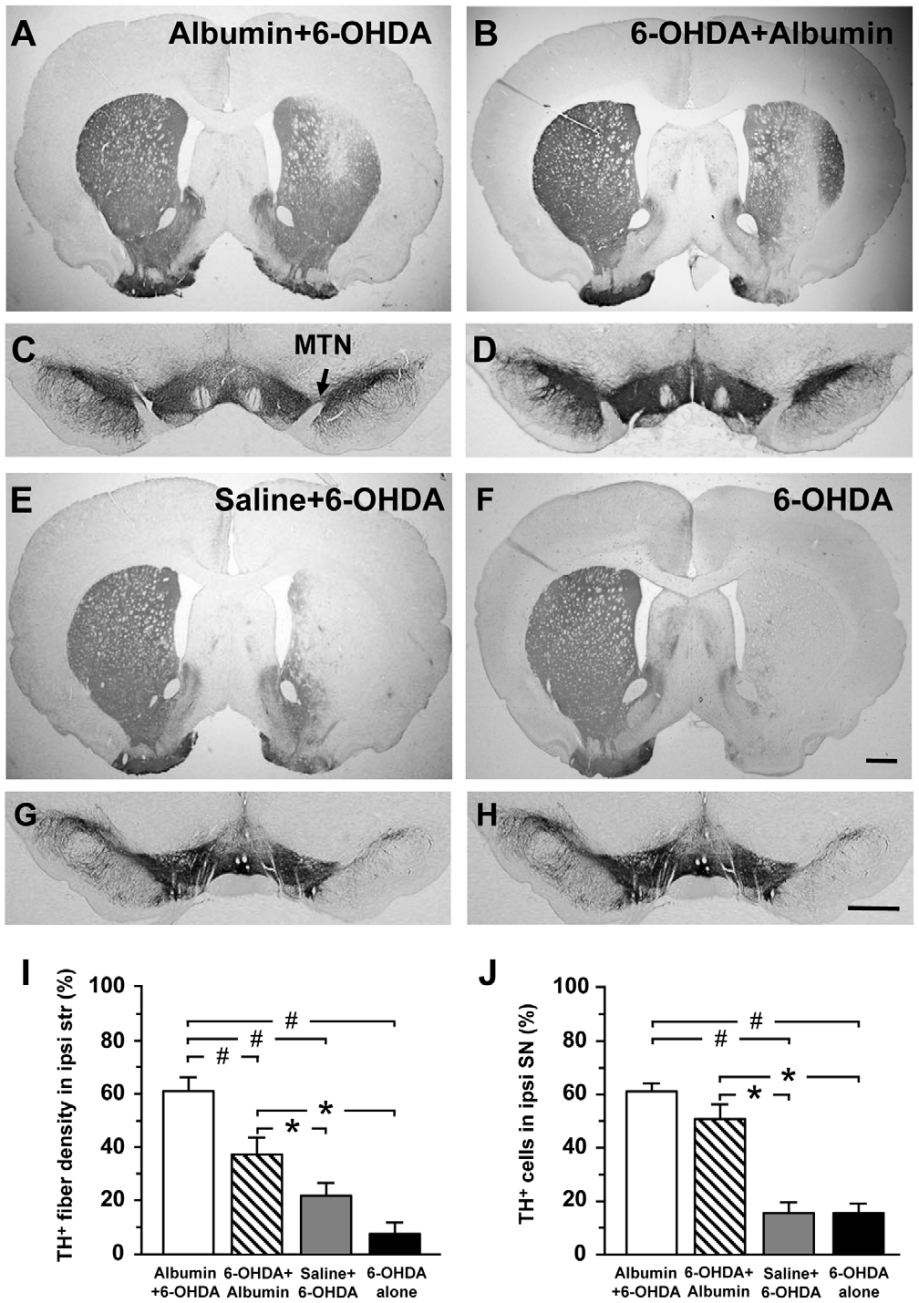
Intrastriatal administration of human albumin protects TH expression of DA neurons in the SNc from 6-OHDA. Photomicrographs were prepared from coronal sections through the striatum (A, B, E, F) and the SNc (C, D, G, H) processed for TH immunocytochemistry in a representative rat of the each group. Scale bars  = 1 mm. (I) Percentage of TH-IR fiber density in the injected striatum relative to the contralateral striatum, and (F) Percentage of TH-IR neurons in the ispilateral SNc, relative to the contralateral SNc for the four different groups. *, # *P<*0.01.

There was extensive immunoreactivity for TH-IR fibers in the right striatum (lesioned striatum) of rats in the Albumin +6-OHDA and 6-OHDA + Albumin groups 10 weeks after 6-OHDA lesion. Only a slight reduction of TH-immunoreactivity was observed in the surrounding areas of needle tracks (Fig. 2A, B). In contrast, a significant reduction of TH-immunoreactivity in the right striatum was observed in the rats of the Saline +6-OHDA and 6-OHDA groups (Fig. 2E, F). The optical densities of TH-immunostaining in the right striatum were 59±4.8% of that seen in the contralateral striatum (internal control) of rats in the Albumin +6-OHDA group and 35.9±6.0% in the 6-OHDA + Albumin group, which were significantly greater than that in the saline +6-OHDA group (20.9±5.1%) and in the 6-OHDA group (7±3.8%) (#, * *P<*0.01) (Fig. 2I).

#### TH-IR cell counts in the SNc

There was intense immunostaining for TH-IR cell bodies in the ipsilateral SNc of the rats in the Albumin +6-OHDA and 6-OHDA + Albumin groups (Fig. 2C, D). In the Albumin +6-OHDA group, the number of TH-IR cells in the ipsilateral SNc of the rats was 61.4±2.9% of the number in the contralateral SNc (internal control), which was significantly greater than that observed in the Saline +6-OHDA group (15.7±3.5%) and in the 6-OHDA group (16.3±3.8%) (Fig. 2G, H) The percentage of TH-IR cells number in the 6-OHDA + Albumin group (50.9±6.1%) was also significantly larger than that in the saline +6-OHDA group and in the 6-OHDA group (#, * *P<*0.01) (Fig. 2J).

### Human albumin protects PC12 cells against 6-OHDA-induced cell death

**Figure 3:**
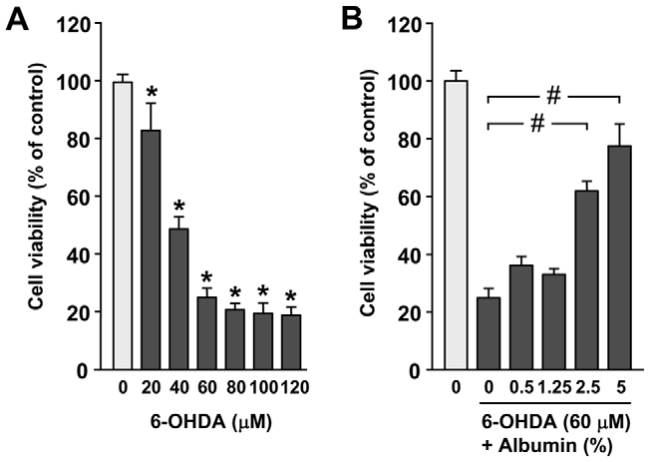
Human albumin protects PC12 cells against 6-OHDA-induced cell death. Cells were treated with 6-OHDA (A) and human albumin (B) at various concentrations for 16 h, respectively, and cell viability was measured by MTT assay and expressed as percentage of untreated control. (C) PC12 cells were treated with 6-OHDA (60 µM for 16 h) in the presence of 5% human albumin. * *P*<0.05 versus untreated cells, # *P*<0.01 versus 6-OHDA-treated cells.

To determine an appropriate dose and treatment time of 6-OHDA and human albumin, we first performed cell viability assays. As shown in Fig. 3A, treatment with 6-OHDA for 16 h resulted in a dose dependent decrease of PC12 cell viability (* *P*<0.05). At 40 µM, 6-OHDA reduced the cell viability by about 50%, and at 60 µM reduction of the cell viability appeared to reach to a plateau level, when compared to the vehicle control. We therefore used concentrations of 6-OHDA ranging 40 to 60 μM in the following experiments. To examine the effect of human albumin alone on PC12 cell viability, PC12 cells were treated with human albumin at different concentrations ranging from 0.5% to 5% for 16 h. Treatment with human albumin alone up to 5% concentration did not affect cell viability of PC12 cells. Human albumin at both doses of 2.5% and 5% significantly attenuated 6-OHDA induced cell death (# *P*<0.01) (Fig. 3B). The data suggested that that human albumin alone does not affect PC12 cell viability, and can prevent from 6-OHDA-induced cytotoxicity in PC12 cells.

### Human albumin inhibits 6-OHDA-induced apoptosis

**Figure 4:**
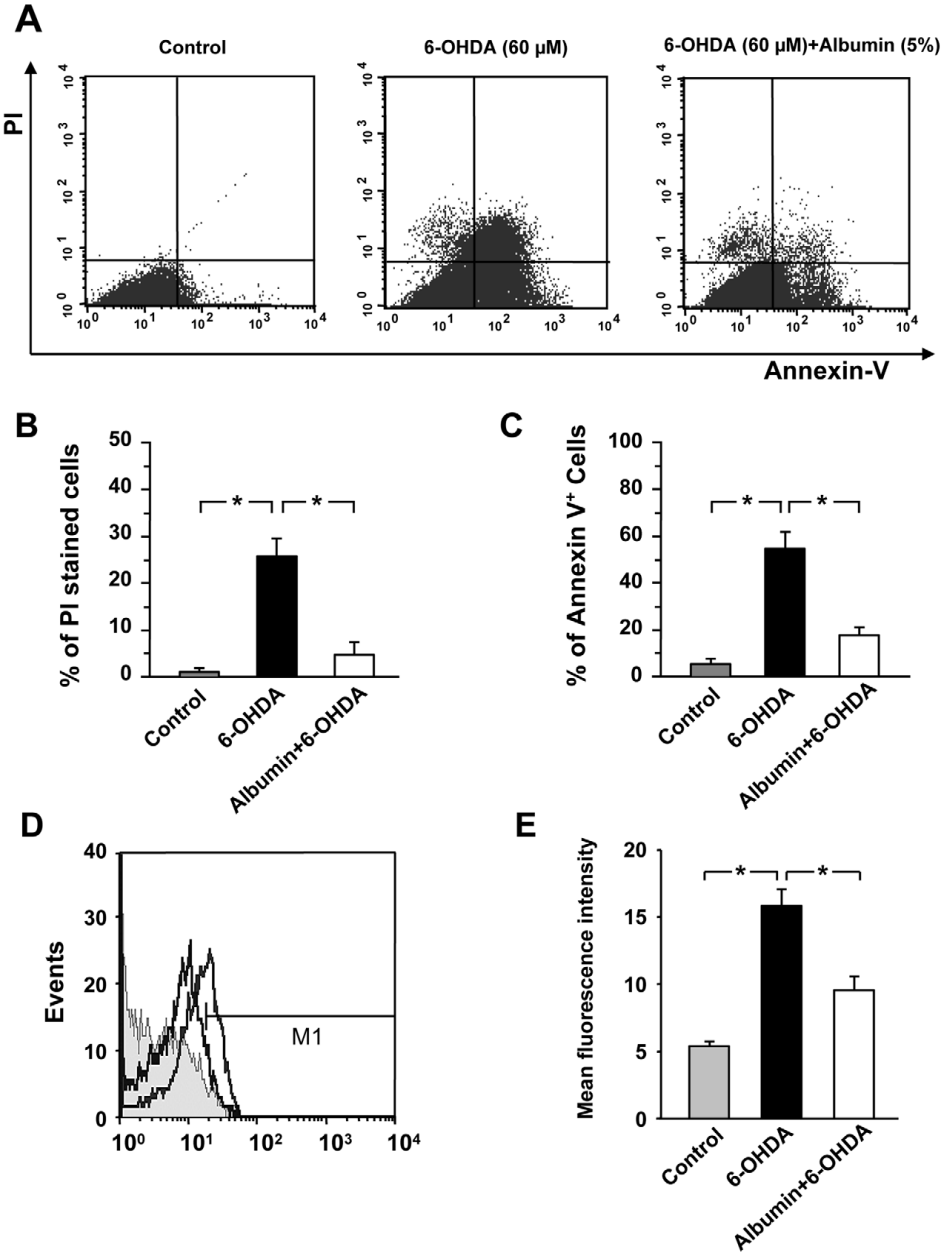
Human albumin inhibits 6-OHDA-induced apoptosis and intracellular ROS formation. (A) Cells were either untreated or treated with 60 µM 6-OHDA for 16 h in the absence or presence of 5% human albumin. The apoptotic cells were labeled with FITC Annexin V and PI for 30 min and analyzed by FCM. Representative data are shown by one of three independent experiments. (B, C) Percentages of apoptotic cells (PI and FITC Annexin V positive) are presented as a bar graph. Data are the mean ± SEM of three independent experiments performed under the same condition. (D) A representative overlay of histograms of ROS in the cultured PC12 cells exposed to 60 µM 6-OHDA with or without 5% human albumin for 2 h, intracellular ROS was stained with DHE for 30 min and analyzed by FCM. The shift to the right of the curve due to increased fluorescence indicates an increase in the intracellular levels of ROS. (E) The mean fluorescence intensity was measured with FCM. The values are presented as the mean ± SEM of three independent experiments. * *P<*0.01 versus untreated cells or 6-OHDA-treated cells.

To determine whether human albumin treatment inhibited 6-OHDA induced apoptosis for PC12 cells, we examined the extent of apoptosis by FCM analysis using double staining with FITC Annexin V and PI (Fig. 4A). Quantification revealed that human albumin treatment significantly reduced the percentages of both PI stained cells (* *P*<0.01) (Fig. 4B) and FITC Annexin V positive cells (* *P*<0.01) (Fig. 4C) in 6-OHDA treated PC12 cells when compared with 6-OHDA alone treated PC12 cells. These results showed that human albumin treatment significantly reduced 6-OHDA-induced apoptosis in PC12 culture.

### Human albumin inhibits 6-OHDA-induced ROS formation

There is growing evidence that the accumulation of intracellular ROS is considered as an important mediator of cytotoxicity, and the initial trigger of apoptotic signaling following 6-OHDA treatment [20]. To determine whether the protective effect of human albumin is associated with inhibition of 6-OHDA-induced ROS production, DHE, an oxidation-sensitive fluorescent dye, was used to measure the intracellular ROS by FCM analysis (Fig. 4D). Quantification revealed that human albumin treatment significantly reduced the mean fluorescence intensity in 6-OHDA treated PC12 cells when compared with 6-OHDA alone treated PC12 cells (* *P*<0.01) (Fig. 4E). These results suggested that human albumin treatment significantly reduced 6-OHDA-induced ROS production in PC12 cultures.

### Human albumin inhibits 6-OHDA-induced caspase-3 activation

**Figure 5:**
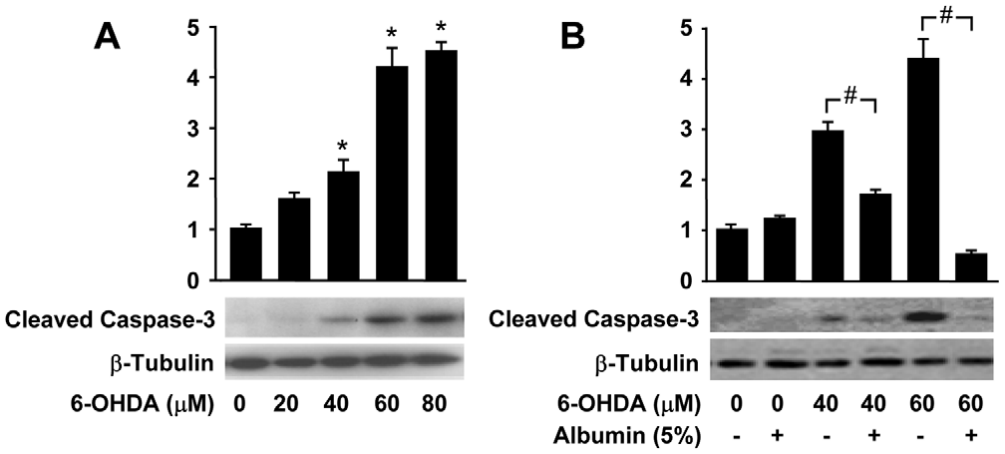
Human albumin decreases 6-OHDA-mediated activation of caspase-3 in PC 12 cells. Western blots for activated caspase-3 of subcellular extracts from PC12 cells exposed to 6-OHDA for 12 h in the absence (A) or presence (B) of human albumin at the indicated concentrations. * *P<*0.01 versus untreated cells, # *P<*0.01 versus 6-OHDA-treated cells at indicated concentrations.

Of all the upstream signals of 6-OHDA induces apoptosis, caspase-3 plays a critical role in the execution of cell death in response to 6-OHDA induced oxidative stress [21]. To further investigate whether human albumin could reduce 6-OHDA-induced caspase-3 activation in PC12 cells, we performed Western blot analysis using antibodies recognizing the active fragments of caspase-3. 6-OHDA treatment increased the levels of cleaved caspase-3 in a dose dependent manner (Fig. 5A). Human albumin treatment significantly reduced 6-OHDA-induced caspase-3 activity in 6-OHDA treated PC12 cells when compared with 6-OHDA alone treated PC12 cells (*, # *P*<0.01) (Fig. 5B).

### Human albumin suppresses 6-OHDA-induced activation of MAPK pathways

**Figure 6:**
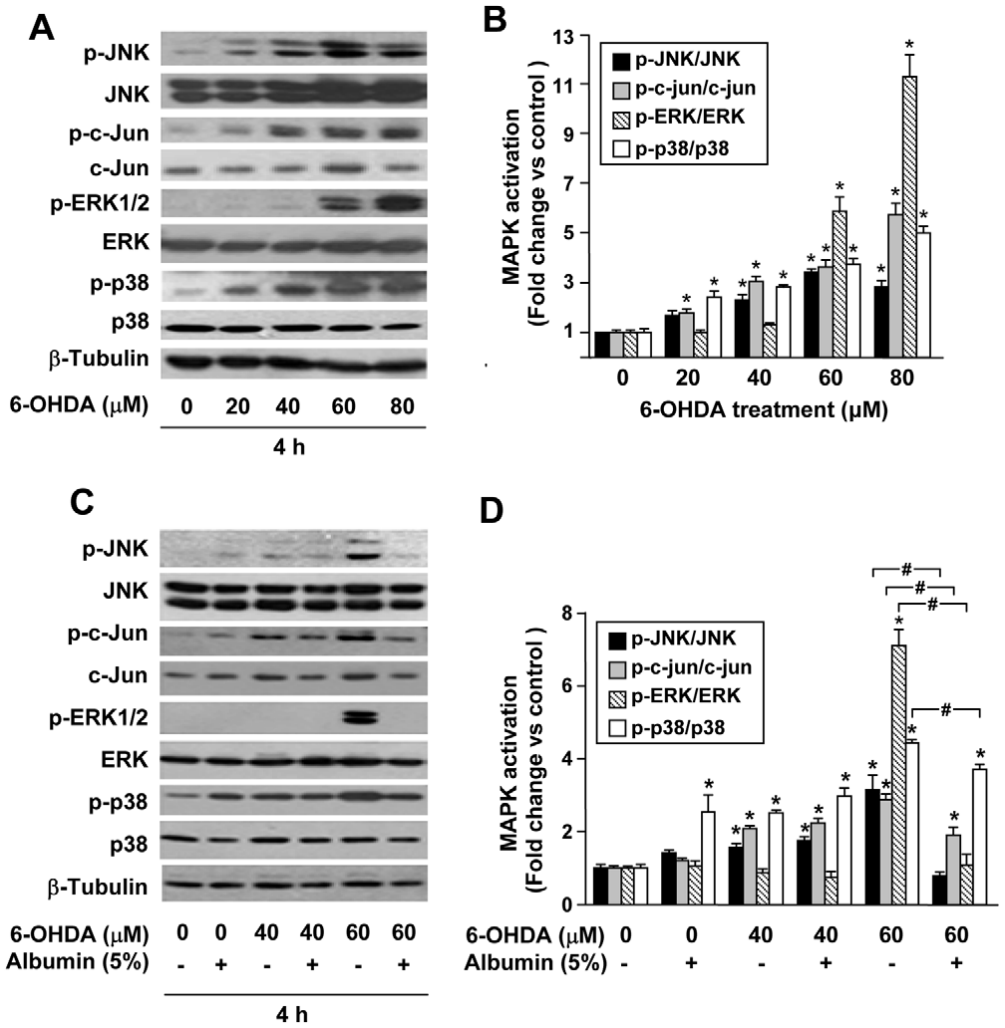
Human albumin inhibits 6-OHDA-induced activation of mitogen-activated protein kinases (MAPK) signaling in PC12 cells. (A) A dose-dependent activation of JNK, c-Jun, ERK, or p38 MAPK by 6-OHDA stimulation. Cells were treated with 6-OHDA (0–80 µM) for 4 h, then the MAPK signaling was detected by Western blot with each specific antibody. (B) The intensity of the bands of phosphorylated MAPK proteins was quantified by densitometry, normalized to that of total MAPK proteins. The fold protein expression was calculated relative to a normalized value of one given to control (untreated) cells and expressed as the mean ± SEM. (C) The inhibition of 6-OHDA-induced MAPK activation by 5% human albumin is shown. MAPK signaling was detected by Western blot after 6-OHDA (40 or 60 µM, respectively) exposure for 4 h, with or without human albumin. (D) The intensity of the bands of phosphorylated MAPK proteins was quantified by densitometry, normalized to that of total MAPK proteins. The fold protein expression was calculated relative to a normalized value of one given to control (untreated) cells and expressed as the mean ± SEM. * *P<*0.05 versus untreated cells, # *P<*0.05 versus 6-OHDA-treated cells at indicated concentrations.

Since activated MAPK family members, including ERK, JNK, p38, may play a role in apoptosis, we examined the phosphorylation of MAPKs by Western blot using anti-phosphorylated antibodies. As shown in Figure 6A, B, treatment of PC12 cells with different concentrations of 6-OHDA (0–80 µM) for 4 h resulted in robust phosphorylation of JNK, ERK1/2, and p38 in a dose-dependent manner. We also examined the phosphorylation of c-Jun, one of substrates of JNK. The increased levels of its phosphorylation also showed a dose-dependent manner. In contrast, the total amount of JNK, c-Jun, ERK and p38 were unchanged during 6-OHDA treatment (Fig. 6A, B). Quantification revealed that the levels of phosphorylation were significantly increased for c-Jun and p38 at 20 µM; for JNK, c-Jun, and p38 at 40 µM; for JNK, c-Jun, ERK and p38 at both 60 and 80 µM when compared with the untreated controls (* *P*<0.05) (Fig. 6B). Next, we examined the role of human albumin on 6-OHDA mediated MAPK phosphorylation. Although 6-OHDA treatment elevated levels of MAPK phosphorylation in PC12 cell cultures, human albumin treatment significantly suppressed 6-OHDA-induced MAPK phosphorylation at 60 µM when compared with 6-OHDA-treated cells (*, # *P*<0.05). (Fig. 6C, D).

### Links between intracellular ROS, MAPK activity, and cell viability: effect of human albumin and other antioxidants

**Figure 7:**
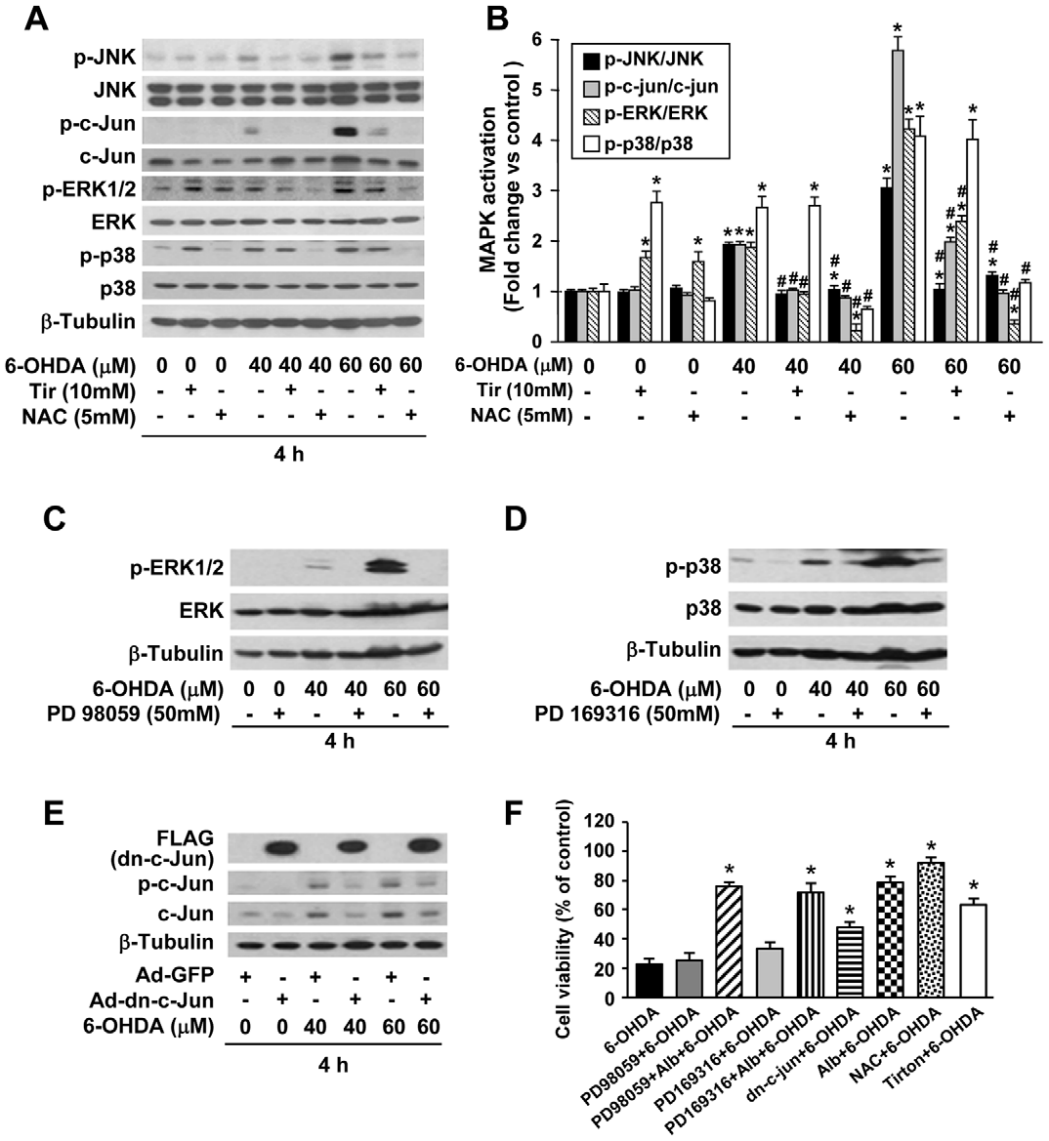
The effect of pharmacological interventions on MAPK activity and on cell viability of PC12 cells exposed to 6-OHDA. (A) Pretreatment of NAC and Tiron attenuated 6-OHDA-induced MAPK activation in PC12 cells. (B) The intensity of the bands of phosphorylated MAPK proteins was quantified by densitometry, normalized to that of total MAPK proteins. The fold protein expression was calculated relative to a normalized value of one given to control (untreated) cells and expressed as the mean ± SEM. (C) PD 98059 (MEK1/2 inhibitor) blocked ERK phosphorylation in PC12 cells exposed to 6-ODHA. (D) PD 169316 (p38 MAPK inhibitor) attenuated the levels of p38 phosphorylation in PC12 cells exposed to 6-ODHA. (E) Transduction of PC12 cells with an adenoviral vector carrying a dn-c-Jun DNA fragment significantly inhibited 6-OHDA-induced c-Jun activation when compared with controls which were transduced with a GFP adenoviral vectors. Top row of the bands showed that PC12 cells were efficiently transduced with a dn-c-Jun-adenoviral vector carrying a Flag marker. (F) PC12 cells were pretreated with various MAPK inhibitors or ROS inhibitors (as described in A–D), and subsequently treated with 6-OHDA (60 µM, 4 h) in the absence or presence of human albumin (5%), and then cell viabilities were measured by MTT assay. * *P*<0.05 versus 6-OHDA-treated cells. * *P<*0.05 versus untreated cells, # *P<*0.05 versus 6-OHDA-treated cells at indicated concentrations.

In the present study, the results suggested that human albumin significantly reduces 6-OHDA-induced production of intracellular ROS and MAPK activation. We next investigated the relationship between ROS and MAPKs in 6-OHDA-induced cytotoxicity for PC12 cells. We observed that antioxidant, NAC dramatically blocked 6-OHDA induced MAPK activation, whereas another antioxidant, Tiron inhibited the phosphorylation of JNK, c-Jun pathway, but not p38 (* *P*<0.05 versus untreated cells, # *P*<0.05 versus 6-OHDA-treated cells at indicated concentrations) (Fig. 7A, B). Results from MTT assay further demonstrated that NAC and Tiron potently suppressed 6-OHDA-induced cell loss of PC12 cells (Fig. 7F). These findings support the notion that activation of MAPK cascades following 6-OHDA treatment might be triggered by accumulation of intracellular ROS.

To dissect the role of individual members of MAPK, we examined the effects of MEK1/2 (upstream of ERK1/2) inhibitor PD98059, or p38 inhibitor PD169316 on phosphorylation of MAPKs and on the cell viability of PC12 cells exposed to 6-OHDA. As shown in Fig. 7C and D, 6-OHDA-induced phosphorylation of ERK1/2 and p38 activity was attenuated by each inhibitor. However, MTT assay showed that PD98059 and PD169316 did not alter cell viability obviously (Fig. 7F). Infection of PC12 cells with Ad-dn-c-Jun resulted in expression of a dominant negative c-Jun, as detected by Western blot (Fig. 7E). Expression of the dn-c-Jun significantly increased cell viability of 6-OHDA treated PC12 cells (Fig. 7E). In addition, MTT assay showed that human albumin acted similar to the protective effects of treatment with dn-c-Jun-adenoviral vector transduction, NAC and Tiron, and significantly increased cell viability of PC12 cells exposed to 6-OHDA (60 µM, 4 h) when compared with controls. This suggests that human albumin protected PC12 cells against 6-OHDA toxicity through an anti-oxidative stress mechanism (* *P*<0.05) (Fig. 7F). In addition, pre-treatment with PD98059, or PD169316 did not affect cytoprotective effects of human albumin on 6-OHDA-induced toxicity in PC12 cell cultures (Fig. 7F). Taken together, these data indicate that the activation of JNK/c-Jun pathway plays a key role in 6-OHDA-induced cell death in PC12 cells. Moreover, human albumin exerts neuroprotection against 6-OHDA-induced cell death by suppression of ROS and MAPK activation in a way similar to antioxidants. Therefore, human albumin may be a potential novel neuroprective agent for treatment of PD.

## Discussion

The present study demonstrates that intrastriatal administration of 1.25% human albumin increases TH-immunoreactivities in the 6-OHDA-injected striatum and enhances the number of TH-positive DA neurons in the ispilateral SNc. Intrastriatal injections of human albumin also lead to the improvement of *d*-amphetamine-induced rotational asymmetry. Furthermore, the study demonstrates that the treatment with human albumin attenuates 6-OHDA-induced PC12 cell death. The results suggest that the protective effect may be mediated by reducing ROS formation, inhibiting caspase-3 activation, and suppressing MAPK signaling. Therefore, human albumin may exert its neuroprotective effects on DA neurons via both antioxidant and anti-apoptosis mechanisms.

Human albumin is a unique multifunctional protein possessing cellular protective properties for a variety of cell types. Human albumin has been shown to protect against apoptosis of chronic lymphocytic leukemia cells [22] and endothelial cells [23], [24], to decrease reactive oxidant-induced neuronal apoptosis, to promote neuronal survival [19], [25], [26], and to increase energy metabolism in primary astrocytes [27]. These studies highlight that human albumin exerts its cellular protective effects through anti-oxidative and anti-apoptotic mechanisms. Human albumin is the plasma's major antioxidant, antagonizing both endogenous and exogenous sources of oxidative stress, exceeding that of vitamin E by 10- to 20-fold [28]. In a series of recent experimental studies, intravenous administration of moderate to high doses of human albumin has been shown to attenuate brain injury in both cerebral ischemic stroke [15], [17], [18], [29], [30] and traumatic brain injury [16], [31]. Here, we demonstrate for the first time that human albumin can prevent nigral TH loss in DA neurons following 6-OHDA lesion and improve behavioral outcome in a rat model of PD. It is known that 6-OHDA causes the loss of TH expression in up to 35% of cells without killing them [32], [33]. Therefore, in this study we have refrained from using the term neuroprotection as we examined only the TH-positive neurons and not the total number of neurons within the SN. However, as oxidative stress plays the key role in initiating cell death process for nigral DA neurons in the 6-OHDA-induced rat model of PD, we speculate that anti-oxidative stress and anti-apoptotic properties of human albumin account for its cytoprotection. In addition, other mechanisms may also contribute to cytoprotection with human albumin. It has been shown that human albumin inhibits endothelial-cell apoptosis [24], and also activates astrocytes and microglia to induce inflammatory responses involved both in cellular injury and repair via activation of MAPK pathways.

Human albumin elicits effects on nigral TH expression of DA neurons following 6-OHDA lesion and may prevent neuronal cell death. It has been demonstrated that albumin can be taken up by cortical neurons [34] and astrocytes [27], [35] via increasing intra- and extra-cellular molecular exchange, and endothelial cells [36] through a receptor-mediated endocytosis. Although we do not have direct evidence that human albumin receptors exist in the cell membrane of DA neurons, immunohistochemical staining showed that human albumin appeared to accumulate in the ipsilateral subtantia nigra after injecting human albumin into the striatum. This suggested that human albumin in the striatum was taken up by either striatal neurons or by the terminals of DA fibers, and transported from the striatum to the subtantia nigra via both anterograde and retrograde transportation pathways. Accumulated human albumin in the substantia nigra may exert local anti-oxidative and anti-apoptotic effects on nigral DA neurons, and protect TH immunoreactiviety against from 6-OHDA lesion. To confirm these direct effects of human albumin, a future study is warranted to examine whether intranigral injection of human albumin is also neuroprotective for nigral DA neurons against 6-OHDA toxicity.

In the Albumin +6-OHDA group, 6-OHDA was injected into the same striatum 1 day after human albumin was injected. Human albumin may neutralize the toxicity of 6-OHDA or interfere with the uptake of 6-OHDA by the terminals of nigral DA neurons in the striatum, leading to ineffectiveness of 6-OHDA lesion. This is the similar issue that was raised in our previous study [37]. To address whether human albumin detoxifies 6-OHDA and reduces its toxicity, we mixed 6-OHDA solution with human albumin in a test tube for 2 hours and separated 6-OHDA from the mixture solution by using a dialysis cassette with molecular weight cut-off of 3500 Dalton. Our preliminary results showed that treatment of 6-OHDA either from the human albumin and 6-OHDA mixture or from 6-OHDA alone solution significantly reduced cell viability of PC12 cells when compared to PC12 cells without treatment of 6-OHDA as assessed with MTT assay. The results suggest that the detoxification of 6-OHDA toxicity by human albumin is minimal. In the present study, the human albumin injection site was chosen 1 mm away from the 6-OHDA injection site based on brain coordinates, and the two injections were made 1 day apart. In addition, dopamine transporter (DAT) immunostaining results did not show significant changes in DAT immunoactivities in the striatum 1 day after intrastriatal injection of human albumin, suggesting that human albumin has no effect on the amount of striatal DAT protein. Nevertheless, the studies on DAT uptake function using other methods, for example, *in vivo* uptake studies with ^3^H labeling, are warranted to examine if human albumin can affect the uptake of 6-OHDA by the terminals of nigral DA neurons. In the present study, human albumin is a foreign protein for recipient rats. We previously reported that intrastriatal injection of human albumin induced local inflammation [37]. Nevertheless, the inflammation seemed not to alter the effects with human albumin. The rats in the Albumin +6-OHDA and the 6-OHDA + Albumin groups that received both human albumin and 6-OHDA in the striatum exhibited both behavioral and morphologic recovery when compared with 6-OHDA control rats.

PC12 cells exposed to 6-OHDA are widely used as an experimental model of PD in vitro [7], [38]. Although some reports highlight the importance of using post-mitotic PC12 cells in the experiments, non-differentiated PC12 cells are still widely used [7], [39], [40]. In the present study, we used non-differentiated PC12 cells as a model system. With regard to the mechanisms responsible to 6-OHDA toxicity, it has been reported that 6-OHDA is oxidized rapidly by molecular oxygen to form the superoxide anion, hydrogen peroxide, and p-quinone. The ROS generated from 6-OHDA initiate cellular oxidative stress and caspase activation, leading to cell death. Here, we examined the neuroprotective capacity of human albumin and investigated the mechanisms responsible for its neuroprotection. In the present study, we clearly demonstrated that 6-OHDA treatment reduced cell viability of PC12 cells, and resulted in apoptosis, ROS formation, and activation of caspase 3 in PC12 cell cultures. All these events were partially reversed by treatment with human albumin, suggesting human albumin could interrupt 6-OHDA-induced oxidative stress process and protect PC12 cells. To confirm this, the effects of antioxidants: NAC and Tiron on cell viabilities of PC12 cells were examined by MTT assay. Human albumin was observed to exert the similar protective effects when compared with antioxidants.

Mitogen-activated protein kinases pathways play a central role in cell death and survival. To evaluate the involvement of these pathways in 6-OHDA-induced toxicity in PC12 cells, we examined the phosphorylation state of MAPKs by Western blot. In agreement with the previous observations [41], we observed that 6-OHDA treatment increased phosphorylation of all three MAPK members including ERK1/2, JNK, and p38 in PC12 cells. The 6-OHDA-induced phosphorylation of MAPKs was attenuated by human albumin treatment, suggesting that human albumin exerted cytoprotective effects via MAPK pathways. To further elucidate the role of individual members of MAPKs in 6-OHDA-induced cell death in PC12 cells, selective inhibitors for each of them were used and their ability to prevent 6-OHDA-induced cell death was examined by MTT assay. We observed that neither the specific MEK inhibitor PD98059, nor p38 inhibitor PD169316 prevented 6-OHDA-induced cell death. Furthermore, the specific blockage of ERK or p38 phosphorylation did not involve in attenuation of 6-OHDA-induced cell death by human albumin. These results suggested that the activation of ERK and p38 play less important roles in the present experimental model. ERK signaling is generally considered a pro-survival pathway [42]. However, some reports have demonstrated that activation of ERK also contributes to cell death [43], [44]. A recent report revealed that ERK activation occurred in a biphasic manner after 6-OHDA exposure in MN9D Cells [45]. ERK family can be stimulated by oxidative stress and affect cellular responses in either a prosurvival or a prodeath manner depending on the kinetics and duration of its activation. For example, pERK tends to enhance survival when activation is rapid and transient [45]–[47], However, delayed and sustained pERK activation has been correlated with cell death [48], [49]. There are conflicting results on inhibition of the p38 signal serving a neuroprotective function. It was reported that p38 MAPK inhibitor afforded significant neuroprotection from LPS toxicity in the neuron-glia mixed culture, but failed to protect DA neurons from 6-OHDA-induced toxicity [50]. However, it was observed that the 6-OHDA-induced cell apoptosis could be effectively prevented by p38 inhibitor SB203580 in human DA neuroblastoma SH-SY5Y cells [11], or by p38 inhibitor PD169316 in MN9D cells and primary cultures of mesencephalic neurons [51]. The differences in experimental conditions, particularly the concentration of 6-OHDA, or cell types used may contribute to these discrepancies.

JNK represents one of the major signaling pathways that is activated by oxidative stress and is considered as an essential molecule in neuronal cell death. Increased JNK activity has been reported in MPTP animal models [52], rotenone neurotoxicity [53], [54], and the 6-OHDA model [39], [55]. JNK-deficient rats exhibited resistance to 6-OHDA- or MPTP-induced injury [56]. It has also been reported that genetically knock down of JNK or intrastriatal administration of JNK inhibitors not only prevent the loss of DA neurons in the SNc but also prevent the loss of DA fibers in the striatum of PD animal models [39], [57], [58]. In the present study, inhibition of c-Jun by viral transduction with a dn-c-Jun adenovirus partially attenuated 6-OHDA-induced cell death, indicating that the activation of JNK, c-Jun pathway is involved in 6-OHDA-induced cell death in PC12 cells.

Substantial evidence suggests that the MAPK pathway is the downstream signaling pathway in ROS-induced apoptosis. To confirm this notion, we examined the effects of ROS scavengers: NAC or Tiron, on 6-OHDA-induced cell death. The similarities observed between the degree of protection against 6-OHDA-induced cell death by human albumin and pre-treatment with NAC or Tiron indicate that human albumin acts via an anti-ROS mechanism. In addition, pre-incubation of PC12 cells with NAC and Tiron inhibited the 6-OHDA-induced activation of MAPK, and protected the cells from apoptosis. These results suggest a link between ROS generation by 6-OHDA and initiation MAPK signaling in PC12 cells. To summarize the results from in vitro experiments, the intracellular generation of ROS by 6-OHDA might be an initial event in 6-OHDA-induced cell death in PC12 cells and activates phosphorylation of MAPK signaling. Thus, better understanding of this signaling pathway should provide insights into new therapeutic targets.

In conclusion, the present study demonstrates that intrastriatal administration of human albumin can prevent nigral loss of TH expression in DA neurons. Although the precise mechanisms responsible for how human albumin does this need to be further determined, the results from *in vitro* experiments suggest that human albumin may exert its cytoprotective effects via anti-oxidative and anti-apoptotic mechanisms. These results provide important implications in the development of antioxidant therapies for PD. As human albumin is used in clinic, human albumin could potentially serve as a therapeutic candidate for PD.

## Materials and Methods

### Materials

Dulbecco's modified Eagle's medium (DMEM) and 0.05% Trypsin–EDTA were purchased from Invitrogen (Grand Island, NY, USA). Horse serum and fetal bovine serum (FBS) were supplied by Hyclone (Logan, UT, USA). Annexin V-FITC Apoptosis Detection Kit I was purchased from BD Biosciences (San Jose, CA, USA). Dihydroethidine (DHE) was purchased from Molecular Probes (Eugene, OR). The MAPK inhibitors, PD98059, and PD169316 were obtained from LC Laboratories (Woburn, MA, USA). Enhanced chemiluminescence solution was from Perkin-Elmer Life Science (Boston, MA, USA). The following antibodies were used for Western Blot: JNK1, phospho-JNK (Thr183/Tyr185), c-Jun, phospho-c-Jun (Ser63), ERK1/2, phospho-ERK1/2 (Thr202/Tyr204), p38, phospho-p38 (Thr180/Tyr182), that were provided from Santa Cruz Biotechnology, (Santa Cruz, CA, USA). Antibody against β-Tubulin, N-acety-L-cysteine (NAC), Tiron, and all other chemicals were purchased from Sigma Chemical Co. (St Louis, MO, USA).

25% human albumin was obtained from Talecris Biotherapeutics, Inc. (NC, USA). Albumin (Human) 25%, USP (Plasbumin®-25) is made from pooled human venous plasma using the Cohn cold ethanol fractionation process. It is prepared in accordance with the applicable requirements established by the U.S. Food and Drug Administration. Plasbumin-25 is a 25% sterile solution of human albumin in anaqueous diluent. The preparation is stabilized with 0.02 M sodium caprylate and 0.02 Macetyltryptophan. The approximate sodium content of the product is 145 mEq/L. Each vial of Plasbumin-25 is heat-treated at 60°C for 10 hours. The production of albumin is known to have contaminants, for example: actate, pyruvate, citrate, fatty acids, glucose, aspartate, and alpha-ketoglutarate [59], [60]. The level of contaminants and their possible biological activity have not been determined in Plasbumin®-25.

### *In vivo* experiments

#### Experimental design

Young adult female Sprague-Dawley rats, weighing 225–250g at the beginning of this experiment, were obtained and housed under a 12h light/dark cycle with *ad libitum* access to food and water in the Animal Core Facility of Capital Medical University (CMU), Beijing, China. All animal procedures were done following the National Institutes of Health Guide for the Care and Use of Laboratory Animals and were approved by the Animal Use and Care Committee of CMU. The number of animals used was the minimize animal required for statistical analysis, and all precautions were taken to minimize animal suffering.

To examine human albumin affect TH expression of nigral DA neurons in a rat model of PD, rats were assigned into the four groups in the experiments. Group 1 (n = 8, denoted as Albumin +6-OHDA): rats received an injection of human albumin into the right striatum and then an injection of 6-OHDA (Sigma-Aldrich, St. Louis, MO, USA) in the same side 1 day later. Group 2 (n = 8, 6-OHDA + Albumin): rats received an injection of 6-OHDA into the right striatum and then an injection of human albumin in the same side 7 day later. Group 3 (n = 7, Saline +6-OHDA): rats received an injection of saline into the right striatum and then an injection of 6-OHDA into the same side 1 day later. Group 4 (n = 7, 6-OHDA): rats only received a striatal injection of 6-OHDA. One week after 6-OHDA lesion, *d*-amphetamine (Sigma-Aldrich, St. Louis, MO, USA)-induced rotational asymmetry was examined for the 6-OHDA + Albumin group. Only the rats with a rotational score of more than 5 rotation/min were selected for further injection of human albumin. At 3 and 10 weeks after the 6-OHDA lesion, *d*-amphetamine-induced rotational asymmetry was examined for all four groups. At the end of the experiment, all rats were sacrificed and brain sections were prepared for tyrosine hydroxylase (TH) immunocytochemistry. The number of DA neurons in the SNc was examined by counting TH–immunoreactive (IR) cells. Striatal denervation was examined by evaluating the optical density of TH-IR fibers in the striatum.

#### Human albumin injection

1.25% human albumin was prepared from 25% human albumin original solution in physiological saline. 2 µl of 1.25% human albumin solution were injected unilaterally into the center of the right striatum of equithesin (3 ml/kg, i.p.) anesthetized rats fixed in a Kopf stereotaxic frame using a 10 µl Hamilton microsyringe (Hamilton Co., Reno, NV, USA) fitted with a steel cannula. Injections were made at the following stereotaxic coordinates: 1.0 mm rostral to bregma; 3.0 mm lateral to the midline; 4.5 mm ventral to the dura; with tooth bar set up at zero. The microinjections were carried out at a rate of 0.25 µl/min. After injection, the cannula remained in situ for additional 4 min before withdrawn. The dose of 1.25% human albumin was chosen based on a previous dose dependent study. In the experiment, intrastriatal administration of 0.25% human albumin was not observed to protect nigral DA neurons against 6-OHDA toxicity in our animal model, whereas, 2.5% human albumin did. By considering that human albumin is a foreign protein to recipient rats and induce immune and inflammatory responses, a moderate dose of 1.25% human albumin was chosen in the present study.

#### 6-OHDA lesions

Intrastriatal injections of 6-OHDA were made as described previously [33]. 15 µg of 6-OHDA, dissolved in 3 µl of 0.2 mg/ml ascorbate-saline were injected into the right striatum of rats at the following stereotaxic coordinates: 0 mm rostral to bregma; 3.0 mm lateral to the midline; 5.0 mm ventral to the dura with the tooth bar set up at zero. The microinjections were carried out at a rate of 0.25 µl/min. After injection, the cannula remained in situ for additional 4 min before being withdrawn.

#### Rotational asymmetry analysis

Rats were given a subcutaneous injection of *d*-amphetamine (5 mg/kg in physiological saline). Rotational asymmetry was monitored for 90 min using an automated rotometer (Rota-count B, Columbus Instruments, Columbus, OH, USA) [61]. Net rotational asymmetry score is expressed as the number of 360° turns per minute. Rotation toward to the lesion side was considered to be positive.

#### Immunohistochemistry

Rats were deeply anesthetized with equithesin (3 ml/kg, i.p.) and transcardially perfused with 0.1 M phosphate-buffered saline (PBS) followed by cold 4% formaldehyde in PBS. The brains were then removed and post-fixed for 4 h in the same fixative, and placed in 20% sucrose in PBS at 4°C until they sank. Sections were coronally cut at 35 µm thickness on a sliding microtome (Leica, McHenry, IL, USA). Four sets of serial sections for each rat were collected into anti-freeze solution using a 24-well cell culture plate and stored at −30°C until further used. The avidin-biotin complex immunoperoxidase technique was used to visualize TH immunoreactivity as described previously [62]. The primary antibody used was against TH (rabbit polyclonal antibody, 1∶500, Pel-Freez Biologicals, Rogers, AR, USA). The secondary antibodies were biotinylated goat anti-rabbit (rat-absorbed) (1∶200, Vector Laboratories, Burlingame, CA, USA). Sections were incubated in ABC solution (Vectastain ABC Elite kit, Vector Laboratories) followed by development with 3, 3′-diaminobenzidine solution (Vectastain DAB kit, Vector Laboratories) to visualize immunoreactivity. To evaluate the specificity of immunostaining, primary antibodies were omitted during the immunostaining as a negative control. Sections were then mounted on superfrost microscope slides (Fisher Scientific, Pittsburgh, PA, USA), dehydrated through ascending graded concentrations of alcohol, cleared in xylene, and coverslipped using DPX mountant (Fluka, Switzerland).

#### Morphological assessment

Immunostained brain sections were examined using light microscopy (Nikon, Japan) with bright-field illumination in a blinded manner. The original coding of the slides, which indicated treatment groups, was covered by opaque tape and the slides were re-numbered. After evaluation, the original codes were revealed. Optical densities of TH-immunostained sections were analyzed using a computer-assisted image analysis system as described previously [63]. All TH-stained sections (12–13 sections per rat) were scanned using a Canon scanner (Canon Inc., Japan). For each section, the right or left striatum was first manually delineated on the screen and the optical density was assessed using NIH image software (Scion Image, Beta 4.0.2). To estimate the TH- immunostaining density, the optical density readings were corrected for nonspecific background density, as measured from the cerebral cortex. The data are presented as ratios to the intact side. TH-IR neurons in the SNc were examined as described previously [33]. Briefly, only sections on which the medial and lateral parts of the SNc were clearly separated by the medial terminal nucleus of the accessory optic tract (MTN) were selected for analysis of nigral cell numbers at rostrocaudal levels of the SNc. Only cells lateral to the MTN were counted. Around four sections were evaluated per rat. Cells were counted using a 10× objective lens. Cells were only counted when they exhibited at least one neurite or had a visible nucleus.

### *In vitro* experiments

To examine the mechanisms underlying neuroprotection of human albumin on DA cells, we used 6-OHDA to challenge PC12 cells (American Type Culture Collection, ATCC, Manassas, VA, USA) as a model system. After treatment with 6-OHDA in the presence and absence of human albumin, PC12 cells were prepared for examining apoptosis, intracellular ROS generation and MAPK signaling. In all *in vitro* experiments, at least three replicas per group were used in each experiment. Data were collected from at least three independent experiments.

#### Cell culture and drug treatment

PC12 cells were maintained in DMEM/F12 medium supplemented with 10% heat-inactivated donor horse serum and 5% FBS at 37°C under an atmosphere of 5% CO_2_ and 95% air. The culture media was changed twice a week. PC12 cells were used for no more than 15 passages. To insult PC12 cells, stock solutions of 6-OHDA (10 mM) were prepared in physiological saline containing 0.15% ascorbic acid and used for all experiments. Cells were treated with 6-OHDA in the absence or presence of human albumin at different concentrations for the indicated time periods. Cells were also pre-treated with the antioxidants: NAC (5 mM) or Tiron (10 mM) followed by exposure to 6-OHDA at 40 µM and 60 µM for 4 h. In addition, cells were pre-treated with MEK1/2 (upstream of ERK1/2) inhibitor PD98059 (50 mM), or p38 inhibitor PD169316 (50 mM) for 1 h, followed by exposure to 6-OHDA at 40 µM and 60 µM in the absence or presence of human albumin (5%) for 4 h.

#### Determination of cell viability

Cell viability was determined by the conventional 3-(4, 5-dimethylthiazol-2-yl)-2,5-diphenyltetrazolium bromide (MTT) reduction assay using a MTT assay kit (Sigma) according to the manufacturer's instructions. Briefly, PC12 cells were plated at a density of 2×10^4^ cells/well in 96-well plates, pre-coated with poly-D-lysine. Cells were treated with the MTT solution (final concentration, 0.5 mg/ml) for 4 h. The medium was removed and 100 µl of DMSO to each well. The formazan dye crystals were solubilized for 30 min, and absorbance at 570 nm was measured with a microplate reader (Molecular Devices, Sunnyvale, CA, USA). Results were expressed as the percentage of MTT reduction, assuming that the absorbance of control cells was 100%.

#### Flow cytometric (FCM) analysis for apoptosis and intracellular ROS generation

Apoptotic cells were assessed by flow cytometry (FCM) using Annexin V-FITC Apoptosis Detection Kit (BD Biosciences) according to the manufacturer's instructions. PC12 cells were plated at a density of 1×10^6^ cells/well into 6-well plates, pre-coated with poly-D-lysine. The treated PC12 cells were digested with 0.25% trypsin + EDTA (Sigma) and centrifuged at 1000 rpm for 10 min, the supernatant was then removed. The cells were washed twice with PBS and fixed by 70% ethanol. The cells were then centrifuged at 1000 rpm for 10 min, washed with PBS for 2 times and adjusted to a concentration of 1×10^6^ cells/ml. To a 0.5 ml cell sample 0.5 ml RNase (1 mg/ml in PBS) (Sigma, USA) was added. After gentle mixing with 5 µl of FITC Annexin V and 5 µl PI, the cells were filtered and incubated in the dark at 4°C for 30 min before FCM analysis.

Intracellular ROS was measured using FCM. PC12 cells were plated at a density of 1×10^6^ cells/well into 6-well plates, pre-coated with poly-D-lysine. The treated cells were collected by pipetting and washed one time with PBS. After DHE (1 µM) was added to cell cultures for 30 min at 37°C, the cells were washed twice with PBS, and adjusted to a concentration of 1×10^6^ cells/ml before FCM analysis. Fluorescence intensity was recorded by using a flow cytometer (Becton Dickinson, San Jose, CA, USA). The mean fluorescence intensity and the percentage of the positive cells were analyzed.

#### Western blot analysis

PC12 cells were plated at a density of 1×10^6^ cells/well into 6-well plates, pre-coated with poly-D-lysine. After treatment, cells were briefly washed with cold PBS. On ice, cells were lysed in the radioimmunoprecipitation assay buffer [50 mM Tris, pH 7.2; 150 mM NaCl; 1% sodium deoxycholate; 0.1% sodium dodecyl sulfate (SDS); 1% Triton X-100; 10 mM NaF; 1 mM Na_3_VO_4_; protease inhibitor cocktail (1∶1000, Sigma)]. Lysates were sonicated for 10 s and centrifuged at 16 000 g for 10 min at 4°C. Protein concentration was determined by bicinchoninic acid assay with bovine serum albumin as standard (Pierce, Rockford, IL, USA). Equivalent amounts of protein were separated on 12% SDS-polyacrylamide gel and transferred to polyvinylidene difluoride membranes (Millipore, Bedford, MA, USA). Membranes were incubated with PBS containing 0.05% Tween 20 and 5% non-fat dry milk to block non-specific binding and were incubated with primary antibodies, then with appropriate secondary antibodies conjugated to horseradish peroxidase. Immunoreactive bands were visualized by using Renaissance chemiluminescence reagent (Perkin-Elmer Life Science). To check the amount of protein loaded, the immunoblots were treated with stripping solution (62.5 mM Tris buffer, pH 6.7, containing 2% SDS and 100 mM β-mercaptoethanol) for 30 min at 50°C and incubated with mouse monoclonal anti-β-tubulin antibody (Sigma) followed by horseradish peroxidase-coupled goat anti-mouse IgG (Pierce). Optical densities of the individual bands were scanned and quantified using NIH ImageJ. The intensity of the bands was normalized by the averaged value of β-Tubulin protein (loading control). The fold protein expression was calculated relative to normalized value of one given to control (untreated) cells as the mean ± SEM.

#### Adenoviral transduction of PC12 cells

Recombinant adenovirus encoding FLAG epitope-tagged dominant negative c-Jun (FLAG-D169) (Ad-dn-c-Jun) was a gift from Dr. Jonathan Whitfield (Eisai London Research Laboratories, University College London, London, UK) [64]. The virus was amplified and titrated as described previously [65]. For experiments, PC12 cells were grown in 6-well plates in the corresponding medium, and transduced with the Ad-dn-c-Jun at 5 of the multiplicity of infection (MOI) for 24 h. Subsequently, the cells were treated with 6-OHDA (40 or 60 µM, respectively) for 4 h. Ad-GFP encoding the green fluorescence protein (GFP) [66] served as a control. Expression of FLAG-tagged dn-c-Jun was confirmed by western blot with antibodies to FLAG.

### Statistical analysis

Data are presented as the mean ± standard error of the mean (SEM). All data were subjected to statistical analysis using StatView software. A two-factor analysis of variance (ANOVA) with repeated measures was used to analyze data for rotational asymmetry for time and treatment effects. A one-factor ANOVA followed by Fisher's post-hoc test was used for group comparisons. Statistical significance was defined at *P<*0.05.
